# Comparing the toxicity and disease control rate of radiotherapy for prostate cancer between salvage settings after high-intensity focused ultrasound therapy and initial settings

**DOI:** 10.1093/jrr/rrac039

**Published:** 2022-07-02

**Authors:** Toshihisa Kuroki, Sunao Shoji, Toyoaki Uchida, Takeshi Akiba, Shigeto Kabuki, Ryuta Nagao, Tsuyoshi Fukuzawa, Yoshitsugu Matsumoto, Tomomi Katsumata, Natsumi Futakami, Tatsuya Mikami, Yoji Nakano, Yuri Toyoda, Tsuyoshi Takazawa, Etsuo Kunieda, Akitomo Sugawara

**Affiliations:** Department of Radiation Oncology, Tokai University Hachioji Hospital, 1838 Ishikawamachi, Hachioji, Tokyo 192-0032, Japan; Department of Urology, Tokai University School of Medicine, 143 Shimokasuya, Isehara, Kanagawa 259-1193, Japan; Department of Urology, Tokai University School of Medicine, 143 Shimokasuya, Isehara, Kanagawa 259-1193, Japan; Department of Radiation Oncology, Tokai University Hachioji Hospital, 1838 Ishikawamachi, Hachioji, Tokyo 192-0032, Japan; Department of Radiation Oncology, Tokai University School of Medicine, 143 Shimokasuya, Isehara, Kanagawa 259-1193, Japan; Department of Radiation Oncology, Tokai University School of Medicine, 143 Shimokasuya, Isehara, Kanagawa 259-1193, Japan; Department of Radiation Oncology, Tokai University School of Medicine, 143 Shimokasuya, Isehara, Kanagawa 259-1193, Japan; Department of Radiation Oncology, Tokai University School of Medicine, 143 Shimokasuya, Isehara, Kanagawa 259-1193, Japan; Department of Radiation Oncology, Tokai University School of Medicine, 143 Shimokasuya, Isehara, Kanagawa 259-1193, Japan; Department of Radiation Oncology, Tokai University Hachioji Hospital, 1838 Ishikawamachi, Hachioji, Tokyo 192-0032, Japan; Department of Radiation Oncology, Tokai University School of Medicine, 143 Shimokasuya, Isehara, Kanagawa 259-1193, Japan; Department of Radiation Oncology, Tokai University School of Medicine, 143 Shimokasuya, Isehara, Kanagawa 259-1193, Japan; Department of Radiation Oncology, Tokai University School of Medicine, 143 Shimokasuya, Isehara, Kanagawa 259-1193, Japan; Department of Radiation Oncology, Tokai University School of Medicine, 143 Shimokasuya, Isehara, Kanagawa 259-1193, Japan; Department of Radiation Oncology, Tokai University School of Medicine, 143 Shimokasuya, Isehara, Kanagawa 259-1193, Japan; Department of Radiation Oncology, Tokai University School of Medicine, 143 Shimokasuya, Isehara, Kanagawa 259-1193, Japan

**Keywords:** high-intensity focused ultrasound (HIFU), radiotherapy, salvage, prostate

## Abstract

The purpose of this retrospective study was to compare the toxicity and disease control rate of radiotherapy for prostate cancer in salvage settings after high-intensity focused ultrasound (HIFU) therapy (HIFU cohort) with those in radical settings (non-HIFU cohort). From 2012 to 2020, 215 patients were identified for this study and 17 were treated in the salvage settings after HIFU. The median follow-up time was 34.5 months (range: 7–102 months, inter-quartile range [IQR]: 16–64 months). Genitourinary (GU) and gastrointestinal (GI) adverse events were evaluated in acute and late periods with Common Terminology Criteria for Adverse Events version 5, and the rates of biochemical-clinical failure free survival (BCFS) and overall survival (OS) were estimated. The cumulative incidence of late GU Grade 2 or greater toxicity after five years was significantly different between the non-HIFU and HIFU cohorts with rates of 7.3% and 26.2%, respectively (*P* = 0.03). Regarding GI Grade 2 or greater toxicity, there was no significant difference between the two cohorts. The 5y-BCFS was 84.2% in the non-HIFU cohort and 69.5% in the HIFU cohort with no significant difference (*P* = 0.10) and the 5y-OS was 95.9% and 92.3%, respectively (*P* = 0.47). We concluded that the possibility of increased late GU Grade 2 or greater should be considered when applying salvage radiotherapy for local recurrence after HIFU.

## INTRODUCTION

In 2020, prostate cancer was the second most frequent cancer and the fifth leading cause of cancer death among men and it is the most frequently diagnosed cancer in more than half of the countries in the world [1]. Mortality rates for prostate cancer have decreased in some countries since the 1990s, which reflects advancements in treatment and earlier detection through screening. The standard primary management for localized prostate cancer includes active surveillance, radical prostatectomy and definitive radiotherapy.

High-intensity focused ultrasound (HIFU) therapy has been studied since the early 1990s as an alternative treatment option for localized prostate cancer. HIFU produces ultrasound waves that are generated by a spherical transducer. The ultrasound energy is focused on a fixed point and the thermal energy destroys the target tissue. Although HIFU has not been compared to other standard approaches in randomized trials and not been included in guidelines for the initial treatment of men with prostate cancer, it has been used as a treatment option for prostate cancer for well-informed patients who requested this method. The reported 5-, 7- and 15-year progression free survival (PFS) rates of HIFU for non-metastatic prostate cancer were 45–88%, 69% and 73%, respectively [2–5].

There has been no sufficient randomized control study on salvage treatment for failure after HIFU, but radiation therapy results have been delivered and reported [6–12]. In these reports, the 3- and 5-year PFS rates for salvage radiation therapy after HIFU were 73.3–77.8% and 64–72.5%, respectively. Although there were differences in the criteria, the reported acute genitourinary (GU) and gastrointestinal (GI) Grade 2 or greater (≥G2) toxicity rates after salvage settings were 0–37% and 4.2–17.6%, respectively, and the late GU and GI ≥ G2 rates were 11–33% and 0–13.3%. [6–12] These toxicity rates were comparable with historical phase 3 trials for prostate cancer as definitive radiotherapy, but there has been no report comparing the frequency of adverse events and disease control rates after radiotherapy between patients who underwent HIFU and those who did not.

Our aim was to compare the toxicity and disease control rate of radiotherapy for prostate cancer between patients in salvage settings after HIFU with those in radical settings.

## MATERIALS AND METHODS

Patients who underwent radiotherapy for non-metastatic prostate cancer at our institution from 2012 to 2020 were included in this study. We excluded those who received radical surgery for pelvic malignancy or radiotherapy, had a history of inflammatory bowel disease, received ultra-hypofractionated radiotherapy and had less than 6 months of follow up. The patients were retrospectively divided into two cohorts: patients who received salvage radiotherapy after HIFU therapy (HIFU cohort) and patients who received radical radiotherapy without preceding HIFU therapy (non-HIFU cohort).

In HIFU cohort, all patients received HIFU by experienced urologists in our institution. The therapy was performed under lumbar anesthesia using a Sonablate 200/500® (SonaCare Medical, USA) from 1999 to 2006, a Sonablate 500 version 4® (SonaCare Medical, USA) from 2005 to 2009, and a Sonablate 500 tissue change monitor® (SonaCare Medical, USA) from 2006 to 2020. The details of the procedures have been described in previous reports [5,13].

A pre-treatment computed tomography (CT) scan (from April 2012 to March 2015, Lightspeed Ultra®, GE healthcare, USA, and from April 2015 to December 2020, Aquillion LB®, Toshiba Medical, Japan) was performed to delineate the prostate, clinical target volume (CTV), planning target volume (PTV) and organs at risk (OARs). All targets and OARs were contoured on a radiation therapy planning system (Eclipse®, Varian Medical Systems, USA). The prostate, seminal vesicle, rectum and bladder were contoured as a solid organ, and the rectum wall and bladder wall were depicted as a structure with an inner wall of 4 mm from the rectum and bladder, respectively. The CTV was set at 0–3 mm from the prostate and up to 17 mm of the seminal vesical origin, which was based on clinical risks by radiation oncologists. The PTV margin was defined as 4 mm from the CTV to the rectum and 8 mm in the other direction.

All patients were treated using a Novalis Tx® (Varian Medical Systems, USA) with a photon beam. The prescribed dose, fractionation and technique have changed with improvement of radiation technology. The prescribed dose per fractions was 2 Gy with conventional fractionation, 3.1 Gy with moderate-hypofractionation. Three-dimensional conformal radiotherapy (3D-CRT) and intensity-modulated radiation therapy (IMRT) were performed. Volumetric modulated arc therapy was used for IMRT in all cases.

In 3D-CRT, preceding kilovoltage portal imaging was performed. Five fixed beams were used with 10MV X-ray. Isocenter of the beams and a reference point was located at the center of PTV. A 100% prescribed dose was delivered at the reference point. In IMRT, we obtained daily kilovoltage portal imaging and weekly cone-beam CT for image-guided radiotherapy with fiducial marker. IMRT was delivered with volumetric modulated arc therapy using 10MV photon beams, and the gantry rotation was one full arc. Patients who received IMRT were delivered a dose covering 50% of PTV volume (D50%).

We retrospectively evaluated baseline characteristics, adverse effects and disease control rates. Baseline characteristics were obtained at the time that initial treatment for prostate cancer was introduced. Recurrence after HIFU was defined based on pathological findings and restaging prior to radiotherapy was performed. Toxicities were assessed from clinician-reports according to the Common Terminology Criteria for Adverse Events version 5 (CTCAE v.5). The largest grades observed during the period were recorded along with the dates. GU toxicity included bladder obstruction, bladder spasm, cystitis, pollokiuria, urethral obstruction, hematuria, urinary incontinence and urinal stenosis. GI toxicity included colitis, constipation, diarrhea, fecal incontinence, hemorrhoids, proctitis and rectal bleeding. Follow-up visits were scheduled every week during radiotherapy and every three months after treatment. We compared categorical data with χ^2^ test statistics; continuous data were compared with Mann-Whitney U test.

We estimated the rates of disease control with biochemical-clinical failure free survival (BCFS) and overall survival (OS). The BCFS rate was defined by any of the following: prostate-specific antigen (PSA) failure with the Phoenix criteria, additional hormonal intervention, clinical local or distant failure, or all-cause mortality.

The time from the start of radiotherapy to the occurrence of each event was assessed. The cumulative incidence of late GU and GI adverse events were analyzed with Gray’s test with death as the competing risk. OS and BCFS rates were determined with the Kaplan–Meier method and analyzed with a log-rank test. To assess risk factors for late GU ≥ G2 toxicity, univariate analyses with Fine-Gray model were performed considering age, initial T stage, initial PSA, initial Gleason score, receiving androgen deprivation therapy (ADT), fractionation type, radiotherapy technique and preceding HIFU therapy. Fine-Gray model was used with death as the competing risk. A *P*-value of <0.05 was considered statistically significant. All statistical analyses were performed using EZR® (Saitama Medical Center, Jichi Medical University, Saitama, Japan), which is a graphical user interface for R® (The R foundation for Statistical Computing, Vienna, Austria) [14]. More precisely, it was a modified version of R® commander designed to add statistical functions frequently used in biostatistics.

## RESULTS

A total of 215 patients were included in this retrospective study. The median follow-up time was 34.5 months (range: 7–102 months, inter-quartile range [IQR]: 16–64 months). Of the total number of patients, 17 patients were given radiotherapy for relapse after HIFU. In the HIFU cohort, 14 patients (82.4%) were treated with whole HIFU, and three patients (17.6%) were treated with focal HIFU. The patient characteristics are summarized in Tables 1 and 2. IMRT was used in 76.5% of the HIFU cohort and in 77.8% of the non-HIFU cohort [(Table 3). Mean prescribed doses were significantly higher in the non-HIFU cohort than the HIFU cohort regardless of radiation technique and fractionation. Therefore, almost all dose volume parameters were also significantly higher in the non-HIFU cohort.

**Table 1:** Patient characteristics in initial settings

	non-HIFU (%)	HIFU (%)	p.value
n	198	17	
Age			
Median [IQR]	75.00 [70.00, 77.00]	73.00 [71.00, 76.00]	0.65
Follow-up months			
Median [IQR]	34.50 [16.00, 64.00]	57.00 [13.00, 64.00]	0.52
Initial PSA (μg/L)			
<10	107 (54.0)	10 (58.8)	0.50
10–20	43 (21.7)	5 (29.4)	
>20	48 (24.2)	2 (11.8)	
Initial T stage			0.04
T1b-T2a	112 (56.6)	15 (88.2)	
T2b-T2c	68 (34.3)	2 (11.8)	
T3a-T3b	18 (9.1)	0 (0.0)	
Initial Gleason score			
≤6	53 (26.8)	4 (23.5)	0.01
3 + 4	37 (18.7)	8 (47.1)	
4 + 3	27 (13.6)	4 (23.5)	
4 + 4	60 (30.3)	0 (0.0)	
9 ~ 10	20 (10.1)	1 (5.9)	
n.a.	1 (0.5)	0 (0.0)	
ADT before RT			
No	43 (21.7)	9 (52.9)	0.01
Yes	155 (78.3)	8 (47.1)	

HIFU: high-intensity focused ultrasound, IQR: interquartile range, PSA: prostate-specific antigen, ADT: androgen deprivation therapy, RT: radiation therapy

**Table 2:** Patient characteristics in the HIFU cohort and re-assessed characteristics prior to radiotherapy

	HIFU (%)
Number of HIFU	
1	5 (29.4)
>2	12 (70.6)
Target of HIFU	
Whole gland	14 (82.4)
Focal gland	3 (17.6)
Gleason score before RT	
≤6	7 (41.2)
3 + 4	0 (0.0)
4 + 3	2 (11.8)
4 + 4	4 (23.5)
9 ~ 10	2 (11.8)
n.a.	2 (11.8)
PSA before RT	
<10	15 (88.2)
10–20	2 (11.8)
>20	0 (0.0)
T factor before RT	
T1b-T2a	15 (88.2)
T2b-T2c	2 (11.8)
T3a-T3b	0 (0.0)

HIFU: high-intensity focused ultrasound, RT: radiation therapy, PSA: prostate-specific antigen

**Table 3:** Fractionation, dose, and technique of radiotherapy

		non-HIFU (%)	HIFU (%)
n		198	17
Conventional fractionation (2Gy/fr)			
Median dose [IQR]		78Gy [72.00, 78.00]	74Gy [70.00, 74.00]
Technique	3D-CRT	44(22.2)	4(23.5)
	IMRT	103(52.0)	10(58.8)
Moderate-hypofractionation (3.1Gy/fr)			
Median dose [IQR]		62Gy [62.00, 62.00]	58.9Gy [58.90, 58.90]
Technique	3D-CRT	0	0
	IMRT	51(25.8)	3(17.7)

HIFU: high-intensity focused ultrasound, IQR: interquartile range, 3D-CRT: three-dimensional conformal radiation therapy, IMRT: intensity-modulated radiation therapy

**Fig. 1:**
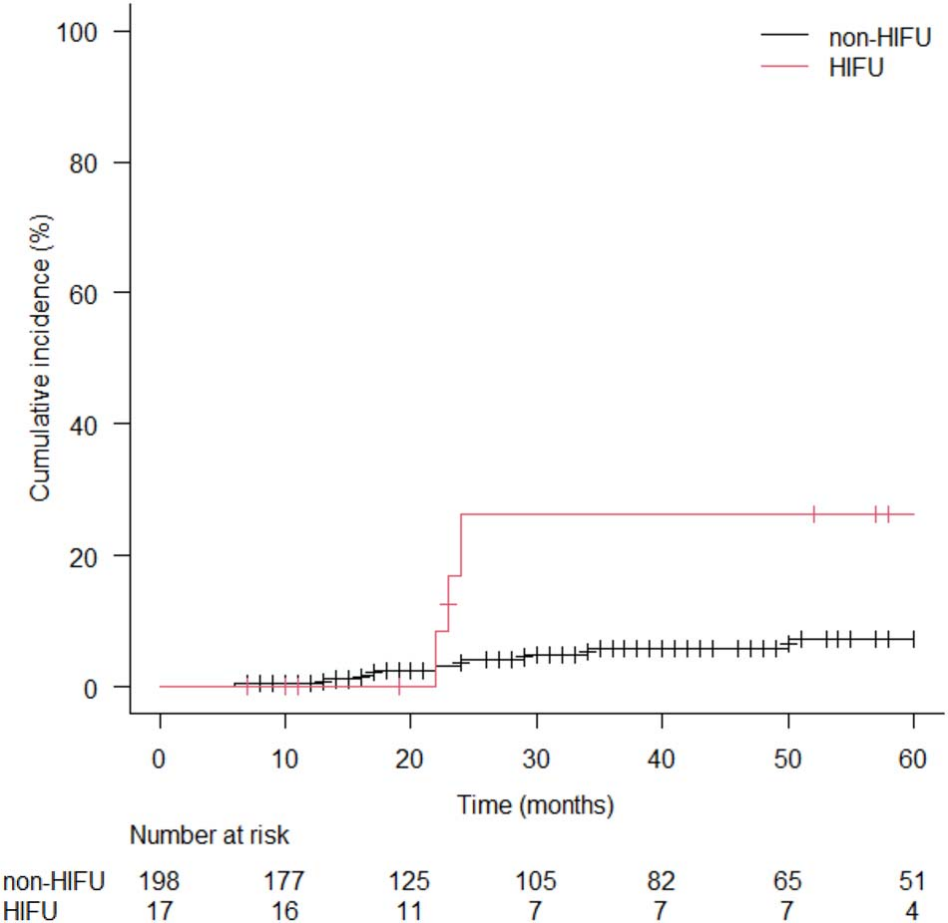
Kaplan–Meier curves for cumulative incidence of late GU Grade 2 or greater toxicity in HIFU and non-HIFU cohorts. HIFU: high-intensity focused ultrasound.

The overall incidence rates of acute and late adverse effects are listed in Tables 4. The acute GU G2 and G3 toxicity rate was 6.1% and 0.5%, respectively, in the non-HIFU cohort. In the HIFU cohort, no patient had GU G2 toxicity, and two (11.8%) patients presented with GU G3 toxicity. Late GU G2 and G3 toxicity rates in the non-HIFU cohort were 2.5% and 2%, respectively, and these rates in the HIFU cohort were 5.9% and 11.8%, respectively. Cumulative incidence of late GU ≥ G2 toxicity after five years was 7.3% in the non-HIFU cohort and 26.2% in the HIFU cohort. Late GU toxicity was increased in the HIFU cohort with statistical significance (*P* = 0.03; Fig. 1). Dose volume parameters were compared in the patients who presented late GU ≥ G2 with who presented late GU G0–1 for each subgroup divided by radiation technique and fractionation. Any dose volume parameter was not significantly different in each subgroup (Tables 5–7).

**Table 4:** GU and GI toxicity (overall incidence)

	GU			GI		
	non-HIFU (%)	HIFU (%)	p. value	non-HIFU (%)	HIFU (%)	p. value
Acute			<0.01			1
Grade 0	64 (31.3)	9 (52.9)		170 (85.9)	15 (88.2)	
Grade 1	123 (62.1)	6 (35.3)		26 (13.1)	2 (11.8)	
Grade 2	12 (6.1)	0 (0.0)		2 (1.0)	0 (0.0)	
Grade 3	1 (0.5)	2 (11.8)		0 (0.0)	0 (0.0)	
Late			0.09			0.70
Grade 0	163 (82.3)	12 (70.6)		163 (82.3)	14 (82.4)	
Grade 1	26 (13.1)	2 (11.8)		25 (12.6)	2 (11.8)	
Grade 2	5 (2.5)	1 (5.9)		3 (1.5)	0 (0.0)	
Grade 3	4 (2.0)	2 (11.8)		7 (3.5)	1 (5.9)	

GU: genitourinary, GI: gastrointestinal, HIFU: high-intensity focused ultrasound

**Table 5:** Dose volume comparison in subgroup who received 3D-CRT

	Late GU G0–1 [IQR]	Late GU ≥ G2 [IQR]	p.value
N	44	4	
age	73.00 [69.00, 77.00]	71.00 [68.00, 74.75]	0.822
Total.dose	72.00 [72.00, 72.00]	72.00 [71.50, 73.00]	0.963
PTV			
Dmean	71.34 [71.03, 71.61]	71.20 [70.57, 72.76]	0.709
Dmax	74.53 [73.49, 75.44]	74.73 [73.81, 76.57]	0.852
D2cc	74.12 [73.01, 74.95]	74.18 [73.25, 75.44]	0.911
Bladder			
Dmean	39.90 [25.56, 47.62]	31.77 [29.17, 40.63]	0.941
Dmax	73.94 [72.79, 75.14]	74.45 [73.13, 76.54]	0.709
D2cc	72.96 [71.90, 74.03]	73.43 [71.79, 75.20]	0.709
D5%	72.06 [70.11, 73.08]	72.70 [70.72, 74.90]	0.654
D10%	70.85 [68.09, 71.95]	71.61 [69.16, 74.34]	0.456
D20%	67.68 [55.52, 69.95]	64.26 [62.87, 67.34]	0.911
D30%	58.66 [38.03, 68.00]	47.97 [47.21, 54.26]	0.911
D40%	48.34 [23.24, 63.89]	33.37 [29.16, 45.14]	0.852
D50%	41.81 [17.26, 53.77]	21.35 [15.16, 37.33]	0.709
Rectum			
Dmean	39.40 [31.53, 43.88]	35.26 [33.60, 36.45]	0.218
Dmax	71.45 [70.60, 72.11]	71.23 [70.05, 74.09]	0.97
D2cc	70.61 [69.60, 71.37]	70.19 [68.98, 72.29]	0.881
D5%	70.43 [68.91, 71.20]	69.97 [68.91, 71.91]	0.941
D10%	69.67 [67.28, 70.65]	69.37 [68.26, 70.24]	0.881
D20%	67.49 [61.95, 68.96]	65.95 [62.63, 67.15]	0.628
D30%	62.16 [48.05, 66.30]	60.62 [56.17, 61.88]	0.502
D40%	52.19 [40.74, 59.56]	46.08 [41.19, 50.43]	0.456
D50%	42.66 [29.55, 47.86]	31.89 [28.48, 35.77]	0.218

3D-CRT: three-dimensional conformal radiation therapy, GU: genitourinary, IQR: interquartile range, PTV: planning target volume, Dmean: mean received dose of the structure, Dmax: maximum received dose of the structure, D2cc: the dose received by 2 cc of the structure, DX%: the dose received by X% of the structure

**Table 6:** Dose volume comparison in subgroup who received conventional IMRT

	Late GU G0–1 [IQR]	Late GU ≥ G2 [IQR]	p.value
n	106	7	
age	75.00 [71.00, 77.00]	73.00 [69.00, 76.50]	0.558
Total.dose	78.00 [78.00, 78.00]	78.00 [75.00, 78.00]	0.134
PTV			
Dmean	79.54 [79.23, 79.78]	78.97 [76.34, 79.73]	0.402
Dmax	84.20 [83.43, 84.93]	84.42 [81.34, 85.52]	0.927
D2cc	81.39 [80.70, 81.96]	81.78 [77.93, 82.37]	0.84
Bladder			
Dmean	33.79 [22.89, 43.59]	40.24 [32.34, 48.62]	0.108
Dmax	82.45 [81.74, 83.31]	83.24 [79.63, 84.83]	0.668
D2cc	80.00 [79.54, 80.57]	80.80 [76.61, 81.35]	0.563
D5%	78.97 [77.58, 79.71]	79.85 [76.16, 80.55]	0.432
D10%	76.77 [68.19, 78.82]	78.64 [74.13, 79.60]	0.186
D20%	62.66 [46.71, 72.17]	70.49 [66.17, 73.33]	0.098
D30%	47.95 [29.86, 60.77]	57.60 [51.50, 62.32]	0.105
D40%	38.00 [13.25, 50.02]	46.00 [34.99, 54.22]	0.163
D50%	27.28 [7.50, 41.43]	39.65 [18.62, 47.66]	0.15
Rectum			
Dmean	37.31 [34.56, 41.32]	37.69 [32.93, 44.08]	0.757
Dmax	79.98 [79.28, 80.61]	79.93 [78.88, 80.38]	0.686
D2cc	75.42 [73.45, 76.20]	74.96 [73.19, 75.69]	0.6
D5%	75.31 [73.93, 75.98]	74.46 [73.14, 75.69]	0.398
D10%	71.25 [68.07, 72.94]	68.43 [65.07, 73.13]	0.588
D20%	59.94 [54.55, 64.79]	52.10 [50.40, 65.44]	0.551
D30%	48.48 [44.28, 55.70]	42.85 [40.65, 56.37]	0.748
D40%	40.56 [36.62, 45.50]	36.88 [34.23, 47.94]	0.877
D50%	32.94 [29.69, 37.87]	32.91 [29.65, 40.73]	0.748

IMRT: intensity-modulated radiation therapy, GU: genitourinary, IQR: interquartile range, PTV: planning target volume, Dmean: mean received dose of the structure, Dmax: maximum received dose of the structure, D2cc: the dose received by 2 cc of the structure, DX%: the dose received by X% of the structure

**Table 7:** Dose volume comparison in subgroup who received moderate-hypofractionated IMRT

	Late GU G0–1 [IQR]	Late GU ≥ G2 [IQR]	p.value
n	53	1	
age	75.00 [72.00, 78.00]	71.00 [71.00, 71.00]	0.351
Total.dose	62.00 [62.00, 62.00]	62.00 [62.00, 62.00]	0.655
PTV			
Dmean	61.07 [60.71, 61.36]	61.58 [61.58, 61.58]	0.102
Dmax	65.92 [65.30, 66.17]	65.95 [65.95, 65.95]	0.822
D2cc	63.94 [63.64, 64.19]	64.24 [64.24, 64.24]	0.352
Bladder			
Dmean	22.14 [13.03, 28.83]	27.04 [27.04, 27.04]	0.461
Dmax	64.89 [64.21, 65.36]	65.68 [65.68, 65.68]	0.211
D2cc	62.98 [62.64, 63.22]	63.70 [63.70, 63.70]	0.149
D5%	60.53 [55.89, 62.35]	62.72 [62.72, 62.72]	0.149
D10%	55.98 [40.09, 60.11]	58.85 [58.85, 58.85]	0.585
D20%	39.91 [24.10, 51.02]	45.58 [45.58, 45.58]	0.63
D30%	30.20 [13.06, 40.20]	36.38 [36.38, 36.38]	0.542
D40%	22.77 [6.52, 31.48]	29.80 [29.80, 29.80]	0.461
D50%	15.86 [4.15, 24.48]	24.08 [24.08, 24.08]	0.423
Rectum			
Dmean	30.12 [27.95, 31.97]	27.12 [27.12, 27.12]	0.29
Dmax	60.66 [59.70, 61.39]	61.56 [61.56, 61.56]	0.262
D2cc	54.29 [52.50, 56.06]	48.23 [48.23, 48.23]	0.102
D5%	54.12 [52.65, 55.24]	48.80 [48.80, 48.80]	0.116
D10%	49.66 [47.44, 51.87]	42.60 [42.60, 42.60]	0.116
D20%	40.94 [38.66, 43.39]	35.15 [35.15, 35.15]	0.168
D30%	34.59 [32.56, 37.38]	30.68 [30.68, 30.68]	0.235
D40%	30.67 [28.21, 32.86]	27.70 [27.70, 27.70]	0.29
D50%	27.24 [25.15, 29.40]	25.42 [25.42, 25.42]	0.461

IMRT: intensity-modulated radiation therapy, GU: genitourinary, IQR: interquartile range, PTV: planning target volume, Dmean: mean received dose of the structure, Dmax: maximum received dose of the structure, D2cc: the dose received by 2 cc of the structure, DX%: the dose received by X% of the structure

**Fig. 2:**
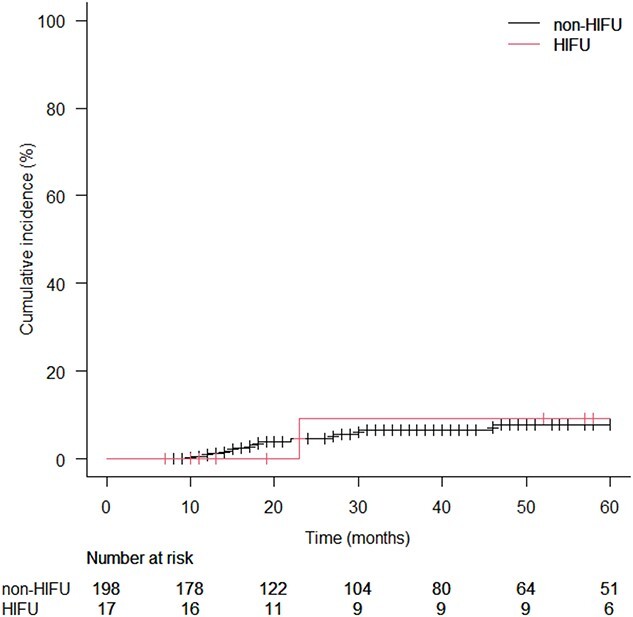
Kaplan–Meier curves for cumulative incidence of late GI Grade 2 or greater toxicity in HIFU and non-HIFU cohorts. HIFU: high-intensity focused ultrasound.

Acute and late GI toxicity rates were low in both cohorts. The late GI ≥ G2 toxicity rate was 4.5% in the non-HIFU cohort and 17.7% in the HIFU cohort (Tables 4). The cumulative incidence of late GI ≥ G2 toxicity at five years was 7.6% in the non-HIFU cohort and 8.4% in the HIFU cohort. There was no statistical significance (*P* = 0.95; Fig. 2).

**Fig. 3:**
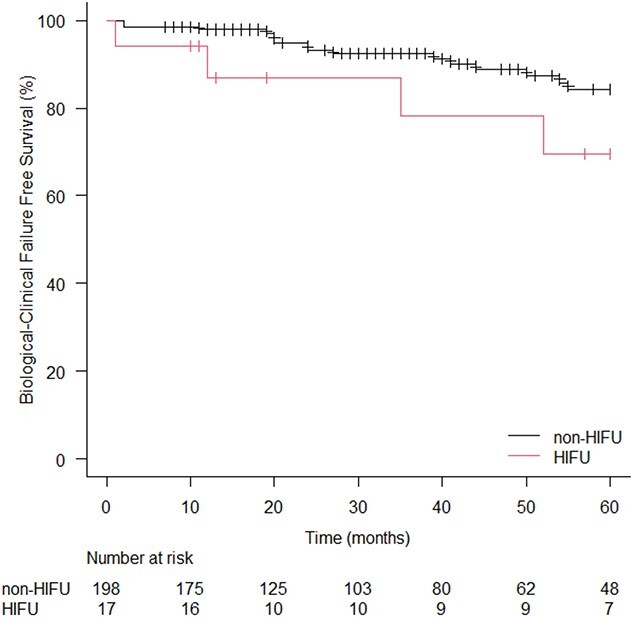
Kaplan–Meier curves for biological-clinical failure free survival rate in HIFU and non-HIFU cohorts. HIFU: high-intensity focused ultrasound.

In the HIFU cohort, two patients had a recto-urethral fistula that required surgery; one had been observed in the period between HIFU and radiotherapy, and the other was observed after salvage radiotherapy. GU and GI Grade 4 or more toxicity was not observed.

**Fig. 4:**
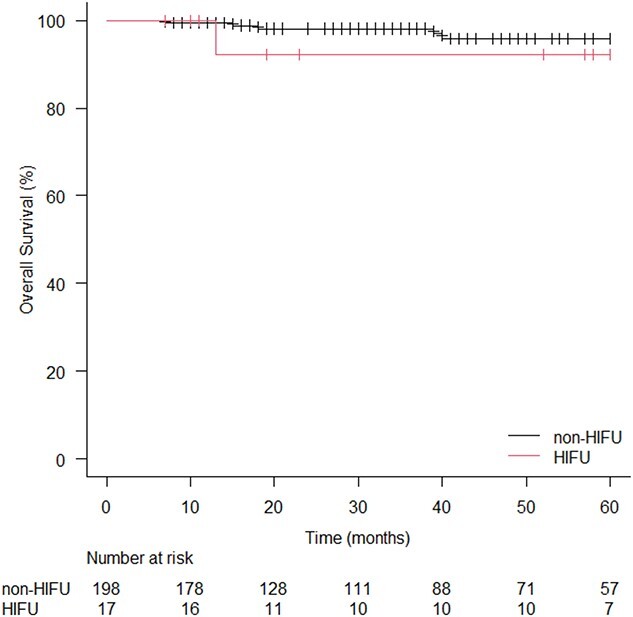
Kaplan–Meier curves for OS rate in HIFU and non-HIFU cohorts. HIFU: high-intensity focused ultrasound.

Recurrence of prostate cancer after radiotherapy was observed in 22 patients (11.1%) in the non-HIFU cohort and in five patients (29.4%) in the HIFU cohort. The 5y-BCFS was 84.2% in the non-HIFU cohort and 69.5% in the HIFU cohort, with no statistical significance (*P* = 0.10; Fig. 3). No patient died from prostate cancer but, overall, six patients died: five patients in the non-HIFU cohort, and the one in the HIFU cohort. The 5y-OS of the non-HIFU and the HIFU cohorts were 95.9% and 92.3%, respectively (*P* = 0.47; Fig. 4).

Preceding HIFU was only variable with significant difference for late GU ≥ G2 toxicity in univariate analysis (HR: 3.93 [95% CI, 1.09–14.15], *P* = 0.04) (Table 8).

**Table 8:** Univariate analysis with Fine-Gray model to identify risk factor for late GU ≥ G2 toxicity

	Univariate analysis	
	HR [95% CI]	p. value
Age		
< 75	(reference)	
≥ 75	0.53 [0.16–1.72]	0.29
Initial T stage		
T1b-T2a	(reference)	
T2b-T2c	0.78 [0.21–2.94]	0.72
T3a-T3b	0.84 [0.11–6.38]	0.86
Initial PSA		
<10	(reference)	
≥10	0.55 [0.17–1.83]	0.33
Initial Gleason score		
≤6	(reference)	
7	0.69 [0.14–3.38]	0.64
≥8	1.46 [0.37–5.73]	0.59
HIFU		
no	(reference)	
yes	3.93 [1.09–14.15]	0.04
ADT before RT		
no	(reference)	
yes	0.48 [0.15–1.55]	0.22
Fractionation of RT		
Conventional	(reference)	
Moderate-hypofractionated	2.77 [0.43–17.71]	0.28
Radiation technique		
3D-CRT	(reference)	
IMRT	0.99 [0.29–3.34]	0.99

GU: genitourinary, G2: grade 2, HR: Hazard ratio, CI: confidence interval, HIFU: high-intensity focused ultrasound, ADT: androgen deprivation therapy, RT: radiation therapy, 3D-CRT: three-dimensional conformal radiation therapy, IMRT: intensity-modulated radiation therapy

## DISCUSSION

Although HIFU is still a controversial focal therapy [15], it has been used nevertheless for well-informed patients who have localized prostate cancer with low to intermediate risk. In the systematic reviews on the use of HIFU as primary treatment in men with prostate cancer [16–18], the reported common adverse effects were erectile disfunction, bladder outlet obstruction, urethral stricture and urinary incontinence. The most frequent adverse event was erectile disfunction and the rate was 0–74%. The adverse events affecting the urinary tract occurred in 0.7–31%, and bladder outlet obstruction occurred in 4–51.5% [17]. He *et al.* [18] reported that the rates of urinary incontinence and erectile dysfunction after HIFU were lower than those after robot-assisted laparoscopic prostatectomy or retropubic prostatectomy. Veereman *et al.* [17] noted that the quality of evidence was low because most of the papers were retrospective case series.

The definition of recurrence after HIFU has varied in reports [16], although the Stuttgart definition with PSA recurrence was proposed [19]. It recommends considering biochemical failure after HIFU when the PSA was elevated by nadir plus 1.2 ng/mL.

Histopathological changes after HIFU were reported by Leenders [20]. They reported that most glands within the HIFU lesion were revealed to have coagulative necrosis by thermal energy, but some glands did not reveal a necrotic sign in intra-HIFU lesions whether malignant or not, and necrosis was found in extra-HIFU lesions such as the proximal seminal vesicles, diaphragm and extra-prostatic fat tissue. They presumed that it might be derived from the heterogeneous circumstances in intra and extra-HIFU lesions; i.e. temperature, exposure time and environmental characteristics within the treated area. When changes occur in the urethra, urogenital diaphragm, seminal vesicles or other lesions, they may lead to adverse events after HIFU. Recently, to reduce such adverse events, some have recommended focal-HIFU [21], which may influence the approach for localized prostate cancer.

Radiotherapy has been reported as a salvage treatment for recurrence after HIFU [6–12]. In these studies, acute GU and GI ≥ G2 rates for salvage settings were reported as 0–37% and 4.2–17.6%, respectively, which were comparable with our study; i.e. both 11.8%. Regarding the rates of late GU and GI ≥ G2 toxicity after HIFU followed by radiotherapy, our results were also comparable with previous reports (Table 9). However, these reports did not compare the rate of adverse events between the HIFU and non-HIFU cohorts. In our study, the 5-year cumulative incidence of late GU toxicity in the HIFU cohort was significantly higher than that in the non-HIFU cohort. This finding suggests that salvage radiotherapy for local relapse after HIFU may increase late GU ≥ G2 toxicity, and more care may be necessary in such salvage settings.

**Table 9:** Overall incidence of late toxicity and oncological outcome after salvage radiotherapy for recurrence after HIFU

First author	Radiation technique	n	Median dose	FUM (median)	Criteria of AE	Late GU > G2	Late GI > G2	Criteria of failure after salvage RT	Results
Riviere J [6]	3D-CRT	100	72Gy	33	CTCAE ver.3	33% ^*1^	2.4% ^*1^	+	5yPFS 72.5%
Pastricier G [7]	3D-CRT	45	71Gy	40	RTOG	11% ^*1^	13.3% ^*1^	++	5yDFS 64%
Munoz F [8]	3D-CRT	24	76Gy	40.3	RTOG	29%	8.4%	Phoenix criteria	3ybDFS 77.8%
Ripert T [9]	3D-CRT	7	74Gy	36.5	RTOG	14% ^*2^	0% ^*2^	Phoenix criteria or the start of ADT	3yDFS 73.3%
Alongi F [11]	IMRT	15^*3^	71.4Gy/28fr	12	CTCAE ver.4	NA	NA	Phoenix criteria	1yDFS 80%
Holtzman AL [12]	Proton	10^*4^	74Gy (RBE)	37	CTCAE ver.4	G3 = 17%^*5^	NA	Phoenix criteria	3ybFFS 77%
Our study	3D-CRT	4	74Gy/37fr	57	CTCAE ver.5	18%	5.9%	Phoenix criteria, the start of ADT or clinical local or distant failure	5yBCFS 69.5%
	Conventional IMRT	10	74Gy/37fr						
	MH-IMRT	3	58.9Gy/19fr						

HIFU: high-intensity focused ultrasound, FUM: follow up months, AE: adverse event, GU: genitourinary, GI: gastro-intestinal, 3D-CRT: three-dimensional conformal radiotherapy, IMRT: intensity modulated radiotherapy, MH-IMRT: moderate hypofractionated IMRT, RBE: relative biological effectiveness, NA: not available, RT: radiotherapy, PFS: progression free survival, DFS: disease free survival, BFFS: biological failure free survival, BCFS: biological-clinical failure free survival, ADT: androgen deprivation therapy, *1: by 1 year, *2: by 2 years, *3: 8 patients treated with pelvic nodes irradiation, *4: 1 patient was recurrence after cryotherapy and 2 patients were treated initial 46Gy with IMRT and Proton boost, *5: only grade 3 toxicity was available, +: three consecutive rises in PSA with a velocity > 0.4 ng/ml per year or PSA >1.5 ng/ml, ++: two consecutive rises in PSA and greater than 1.5 ng/mL, or the administration of ADT

Irradiated area for prostate cancer generally includes part of organs surrounding the prostate, such as urethra, rectum, seminal vesicle and bladder. The irradiated tissues develop microvascular disorders, a failure of the regenerating epithelium, fibrosis and necrosis, which leads to adverse events [22–24]. Although the histopathological changes after radiotherapy are different from those after HIFU, both therapies can cause similar adverse events. We considered it possible that they may affect each other when treated with both therapies, and our results suggesting that radiotherapy increased the cumulative incidence of late GU toxicity may support this hypothesis.

In this study, the 5y-BCFS and 5y-OS of the HIFU cohorts were 69.5% and 92.3%, respectively, and of the non-HIFU cohorts were 84.2% and 95.9%, respectively. The 5y-BCFS of the HIFU cohort seemed to be lower than that of the non-HIFU cohort, but there was no statistically significant difference (*P* = 0.10). The failure rates were almost identical to previous studies in both non-HIFU and HIFU cohorts (Table 9), although they applied different definitions of failure. We consider that certain oncological outcomes may be expected with salvage radiotherapy after HIFU.

Its retrospective nature and the small patient cohorts limited this study. We assume that the reason for which patients had good oncological control after HIFU and that if relapse was diagnosed, some might have been treated with another modality, such as hormonal therapy or prostatectomy.

The patient characteristics initial T stage, initial Gleason score and ADT before and during radiotherapy were different in the two cohorts. This is presumably because HIFU therapy had been applied for relatively low-risk prostate cancer and intermediate to high-risk patients had received ADT based on previous findings. Lawton *et al.* [25] reported the use of hormonal intervention before or during radiotherapy significantly decreased the rate of late GU and GI G3 or greater toxicity and speculated that ADT might have some protective effects. Though the reason is still unknown, this may have affected the results of this study, but there is no evidence comparing the G2 or greater with or without ADT.

Only clinician-reported toxicity reports were available and there was no score-based assessment, such as the International Prostate Symptom Score or the International Index of Erectile Function. For this reason, we could not evaluate the severity of adverse events with each symptom such as urethral stricture, dysuria and urinary incontinence. We consider this to be a topic for future research.

Our univariate analysis showed that preceding HIFU was the only statistically significant variable for late GU ≥ G2 toxicity. We think the finding have an important implication because there have been no studies assessing risk factors for late toxicity with and without preceding HIFU and we will plan prospective study to evaluate this.

Based on these results, when applying salvage radiotherapy for local relapse after HIFU, the possibility of increased late GU ≥ G2 should be considered, although the disease control rates were comparable with radiotherapy as initial treatment.

## Notes

(Received Month Day, 2022; revised Month Day, 2022; Editorial Decision Month Day, 2022)
